# Frequency and tendency of malaria in Colombia, 1990 to 2011: a descriptive study

**DOI:** 10.1186/1475-2875-13-202

**Published:** 2014-05-29

**Authors:** Alexandra Porras Ramirez, José Israel Galindo Buitrago, Juan P Pimentel González, Andres Herrera Moráles, Gabriel Carrasquilla

**Affiliations:** 1 Fundación Santa Fe de Bogotá Centro de Estudios e Investigación en Salud - CEIS, Carrera 7 B # 123-90, Piso 3, Bogotá, Colombia

**Keywords:** Models, Statistics, Malaria, Biological, Colombia, Epidemiology

## Abstract

**Background:**

Malaria is a serious health problem in Colombia. This paper intends to analyse the frequency and tendencies of the disease in Colombia over the last 22 years. The researchers used the Box-Jenkins (ARIMA) methodology for the analysis of time series.

**Methods:**

This descriptive study was done retrospectively by using the morbidity records of the Ministry of Health and of the System for the Monitoring of Public Health (SIVIGILA). The information about the population was obtained from the National Administrative Department of Statistics (DANE). The incidence rate according to age and sex was calculated from 1990 to 2011. Also, the Annual Parasite Index (API) for *Plasmodium falciparum* and for *Plasmodium vivax* was calculated. The mortality rates per year, from 1990 to 2011, were determined. Finally, the Box-Jenkins (ARIMA) methodology was used for the analysis of time series, grouped weekly. Information for ARIMA modelling was used from the year 2001.

**Results:**

The total number of reported cases from 1990 to 2011 was 2,964,818 cases with an annual average of 134,764. In the period from 2001 to 2005 and from 2006 to 2011 a significant decrease of annual cases was observed. In general, a predominance of *P. vivax* over *P. falciparum* was observed*.* With respect to the API, it must be noted that there were peaks in 1994 in the departments of Guainía and Guaviare, and in 1998 in Guaviare and Chocó. The department of Antioquia showed a tendency towards a decrease of the API through the years.

In the time series model there were no statistically significant seasonal patterns for the total number of cases of malaria. However, for *P. falciparum* the number of cases was statistically significant. Lastly, between 1990 and 2009, there were 1,905 deaths caused by malaria in Colombia with a significant tendency towards a decrease in deaths over those years. *Plasmodium falciparum* was more lethal than *P. vivax.*

**Conclusions:**

In Colombia, the transmission of malaria occurs in an endemic and epidemic context, which keeps an unstable endemic transmission pattern. Several factors specific to a country such as Colombia encourage the dissemination and permanence of the illness.

## Background

Malaria is a serious health problem in Colombia. In the country, 85% of the rural territory that is below 1,600 metres above sea level has climatic, geographic and epidemiological conditions that are suited for the transmission of the disease. Consequently, it is estimated that between 18 and 30 million people are at risk of becoming sick or of dying of malaria [1-3]. Of the total population exposed, 2,646,075 (8.8%) people live in high risk zones, concentrated in 168 municipalities with an Annual Parasite Index (API) of 21.2/1,000 inhabitants and an annual malaria index for *Plasmodium falciparum* (AFI) of 5.7/1,000 inhabitants. On the other hand, around 10,982,664 people live in medium risk zones, with an API of 4.5/1,000 inhabitants and an AFI of 1.9/1,000 inhabitants and 16,294,035 people live in low risk zones, with an API of 0.1/1,000 inhabitants and an AFI of 0.04/1,000 inhabitants [1,4,5].

This work aimed to analysing the frequency and the tendency of malaria in Colombia in the last 22 years. Its purpose is to contribute to the understanding of the impact of interventions developed by governmental programmes at national and state level and by programs funded by international organizations, such as the Global Fund to Fight AIDS, Tuberculosis and Malaria. The results of the study should provide information for the public health decision-making [6]. Likewise, this paper constitutes a specialized epidemiologic surveillance activity that, in many cases, helps to alert about its future importance [6].

## Methods

A descriptive study was carried out to determine the tendency of malaria in Colombia between 1990 and 2011. The information of malaria cases was obtained from the morbidity records that the vector control programme of the Ministry of Health gathered for the years 1990 to 1997, and from the Public Health Surveillance System (SIVIGILA) of the National Institute of Health for the years 1998 to 2011. The information about the population was obtained from the population projections made by the National Department of Statistics (DANE) for the years 1990–2011 [7].

Incidence rates were calculated and expressed in cases per 100,000 people. In the case of deaths, the data was obtained from the records of individual death certificates that were consolidated in the mortality databases of DANE.

Specific mortality rates according to age and sex groups were calculated, and they were expressed in number of deaths per 100,000 people per year. Time series were grouped weekly. With the purpose of obtaining comparative measures throughout time, the ratio of incidence was calculated for the series by dividing the number of malaria cases reported by SIVIGILA by the corresponding population.

The Box-Jenkins (ARIMA) methodology [8] was used for the time series analysis, which considers each of the temporary series as the performance of an underlying stationary stochastic process.

## Results

### Morbidity

**Figure 1:**
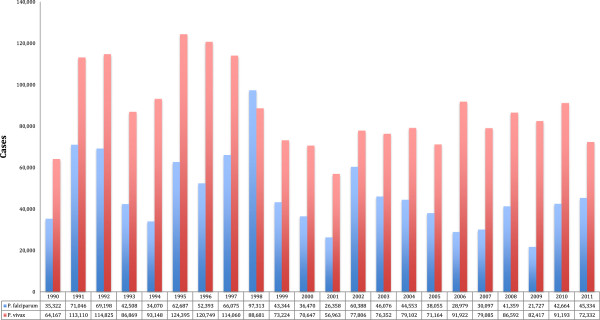
**Frequency of the number of malaria cases according to parasite species in Colombia, 1990 to 2011.** Source: Instituto Nacional de Salud. SIVIGILA, 1990 to 2011. In the analysed period (1990–2011), the total cases of malaria cases was 2,851,285, 1,841,547 were for *P. vivax* equivalent to 64.6%, followed by *P. falciparum* with 990,331 equivalent to 34.7% and mixed forms of malaria with 19,407 equivalent to 0.7%.

The total number of reported cases for the period was 2,964,818, with an annual average of 134,764. Cases reported to SIVIGILA have a non-uniform tendency, which could show a possible seasonal factor of the data (Figure 1). The information on malaria may be broken down according to periods of time. It was found that for the 1990–1995 period, the annual average of notified cases was 151,891, with an average *P. falciparum – Plasmodium vivax* relation of 34% *versus* 66%, respectively.

The API observed in 13 departments with 90% of malaria cases was 450.3 per 1,000 inhabitants between 1991-2011. Seven out of them had an API of 643.2 per 1,000. There are peaks of API in Guainía and Guaviare for the year 1994 and in Guaviare and Chocó for the year 1998. The department of Antioquia has shown a defined tendency towards the decrease of the API through the years, although it has shown a peak, again, in the years 2000 and 2009 with an average number of cases of 46,326 per year.

**Figure 2:**
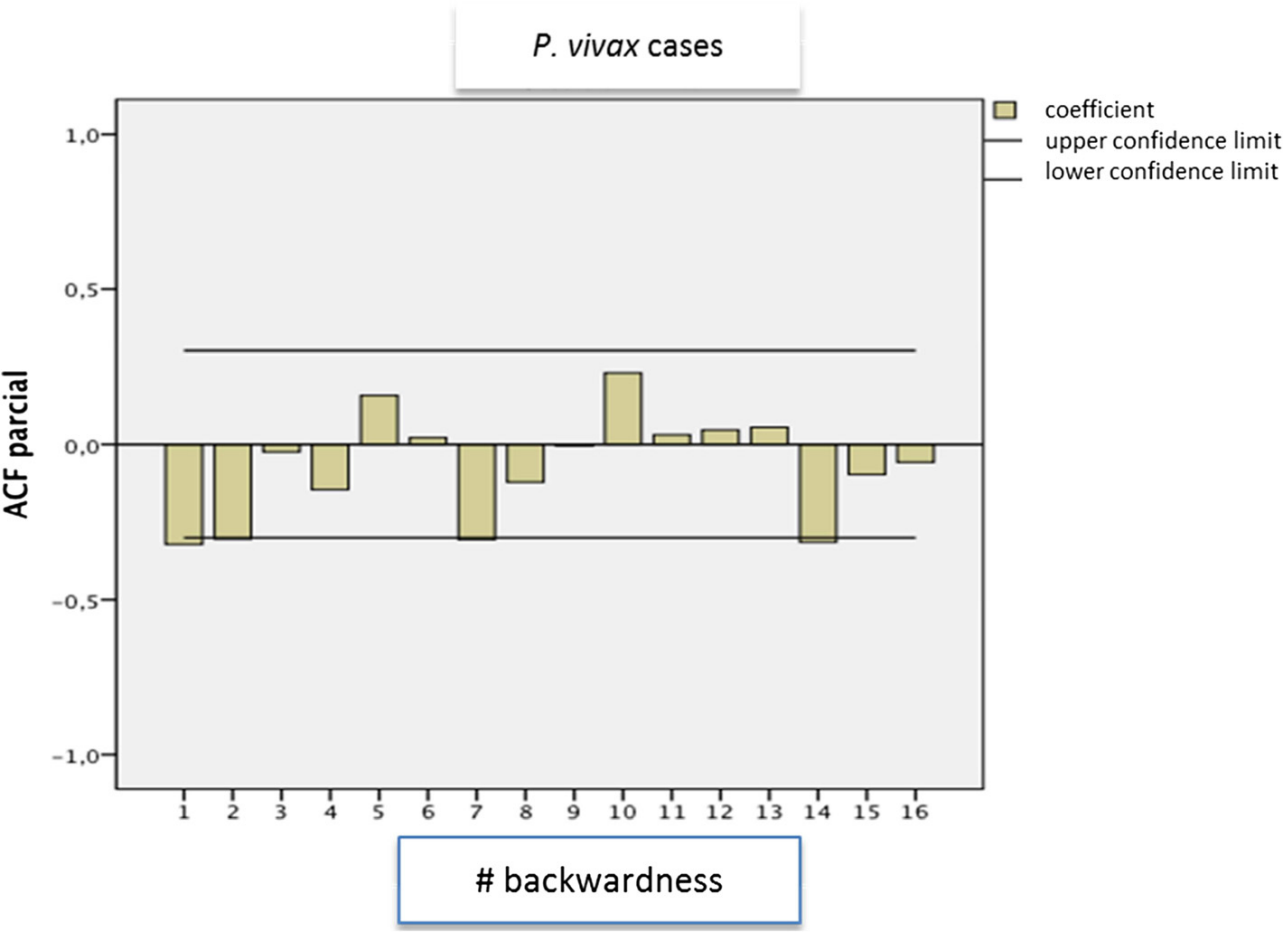
**Time series for *Plasmodium vivax *cases per epidemiologic week in Colombia from 2001 to 2011.** Source: Instituto Nacional de Salud. SIVIGILA, 1990 to 2011. Legend: Cases of vivax 733,389. Coordinates: X = No. of weeks. Y = Autocorrelation Function.

In the time series model, no significant statistical seasonal patterns were found for the total malaria cases. For *P. vivax,* a peak is observed every five weeks in a year that is considered endemic and every 5, 6, and 7 weeks in an epidemic year (p = 0.151) (Figure 2).

**Figure 3:**
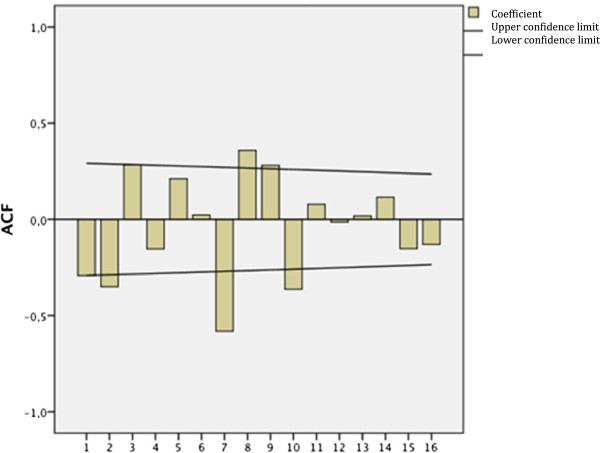
**Time series for cases of *Plasmodium falciparum *per epidemiologic weeks in Colombia from 2001 to 2011.** Source: Instituto Nacional de Salud. SIVIGILA, 1990 to 2011. Legend: Cases of Falciparum 364,690 Coordinates: X = No. of weeks. Y = Autocorrelation Function.

For *P. falciparum,* it has been observed that there is a peak every three weeks in one epidemic year, and every three to seven weeks in an endemic year. These cases are statistically significant (p = 0.04) (Figure 3). This would indicate that after the first increase in cases outside the expected, increasing cases increase every 3 to 7 weeks matching the natural history of the disease and the habits of the vector (Figure 3). In other words, the graphics on the autocorrelation function of the series (auto-correlation and auto-correlation part, respectively), a seasonal component reflected on the frequencies of *P. falciparum*. This fact corroborates the presence of a seasonal cycle of close to seven weeks in epidemic year duration.

### Mortality

**Figure 4:**
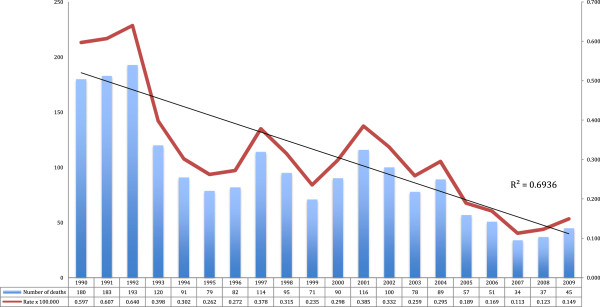
**Frequency of mortality from malaria in Colombia, 1990 to 2009.** Source: Death certificates and mortality databases. DANE 1990-to 2009.

Between 1990 and 2009, 1,905 deaths caused by malaria were registered in Colombia, 1,029 (54%) out of them were men. However, this is not a statistically significant difference. Between 1990 and 2009, a statistically significant tendency towards the decrease in number of deaths (R^2^ = 0.69) was observed. It went from 180 to 45 in this period (Figure 4).

The parasite was identified only in 400 (21.1%) deaths, *Plasmodium falciparum* was identified in 81.0%, *P. vivax* in 6.5%, *Plasmodium malariae* in 2.5%, and ‘other parasite’ species in 10.0%. The crude rate of mortality by malaria for the period of study was 0.84 deaths per 100,000 inhabitants, 0.81 for men and 0.66 for women. The age-adjusted rate was 0.85 per 100,000 inhabitants, 0.89 for men and 0.71 for women.

The tendency of the mortality rates from malaria was not the same between men and women or for the study period. From 1990 to 1995, a tendency towards the decrease of the percentage of annual change was observed. The percentage of annual change was 88.3%, which is statistically significant. For the period from 1995 to 2000, as well as for the period from 2000 to 2005, a stable tendency with an average of 88.5 and 88.3 annual deaths is shown. However, for the 2005 – 2009 period, an important decrease with an annual average of 41.7 annual deaths is observed.

## Discussion

In Colombia, malaria transmission is produced in an endemic and an epidemic context, which keeps an unstable endemic transmission around the country. Colombia's geographical features favor the existence of diverse thermal levels and an abundance of anopheline vectors. Added to the above, the permanent migration of population groups favors the dissemination and permanence of the disease. Difficulties in accessing the health system and its inherent flaws further worsen the panorama [9].

As per the study results, the cases reported to SIVIGILA have a non-uniform tendency that could show a possible seasonal factor of the data. However, when data are broken down in periods of five years, in general a tendency towards the decrease of cases over time (using as reference the 1990–1995 period) is observed. It is taken into account that the only period that did not show a statistically significant decrease was 1996 to 2000 (t student = 2.37, p = 0.982). Such decrease over the decade, according to the opinion of coordinators of the malaria programme, was partly due to the fact that, during the transition period (1994–1998) of the decentralization process, there were important deficiencies in data collection that led to underreporting of cases [10].

However, the remaining periods (2001 to 2005 and 2006 to 2011) did show a significant decrease of annual cases (t student = 34.5, p = 0.001 and t student = 43.2 p = 0.000 respectively). This result coincides with the reports of the World Health Organization that show a significant reduction of the reported malaria cases in the Americas during the decade, going from 1,18 million of reported cases in 2000 to 526,000 cases in 2009 [11]. In the case of Colombia, a study conducted by Padilla and collaborators reports similar results and concludes that in the 2000–2009 decade, a decreasing tendency of the malaria cases was observed. It went from 144,432 in the year 2000 to 79,252 cases in the year 2009 [9]. Colombia, Brazil and Guyana are, therefore, among the group of Latin American countries that had a reduction of malaria cases from 25% to 50% in the above-mentioned decade [11].

There is a predominance of *P. vivax* over *P. falciparum*. These findings are consistent with other studies carried-out in Colombia, which report that, since the year 1974, *P. vivax* has become predominant. The study results are also congruent when stating that, in the year 2010, there was an increase of cases by *P. falciparum* that impacted the *P. falciparum/P. vivax* ratio [9].

In order to predict with some degree of reliability some aspects related to the malaria epidemiology, the time series models such as the one developed in this study have been utilized, successfully, in other geographic areas. It is worth highlighting the studies carried-out by Gomez-Elipe *et al.*[12] who developed an ARIMA model. It seeks to find the relations between the monthly notification of malaria cases and the environmental factors, such as rain and temperature, in Burundi.

In this work, no statistically significant seasonal patterns were found for the total number of cases of malaria. However, the figures of the autocorrelation functions of the series (auto-correlation function and partial auto-correlation, respectively), showed a seasonal component in the frequencies of malaria for *P. falciparum*. This fact corroborates the presence of a seasonal cycle that lasts close to seven weeks in an epidemic year. It is important to take into account that the ARIMA models for time series analysis have also been used, successfully, for the prediction, in an isolated manner, of the number of cases in endemic areas worldwide [13]; therefore, these results are pertinent for planning and managing prevention programmes and local control.

With regards to mortality by malaria, findings of this work show, also, a statistically significant tendency towards the decrease in the number of deaths in Colombia (R^2^ = 0.69). This result coincides with similar studies conducted in this country, such as the one described by Chaparro and Padilla [14], who found that the mortality rate adjusted by age was 0.74 deaths per 100,000 inhabitants in the period between 1979 and 2008. In the present study, the mortality rate adjusted by age was slightly higher (0.85 per 100,000 inhabitants). This was possibly because Chaparro and Padilla’s study considered the 80’s decade, time in which there was a marked descent when compared with the 90’s decade. In general, there has been proof of a decreasing tendency of mortality by malaria in the region of the Americas. There was an important decrease of the disease in countries such as Brazil and Peru [15]. In Colombia, diverse initiatives like the decentralization process, the incorporation of the global malaria control strategy, the use of rapid diagnostic test, the introduction of more effective therapeutic plans, and the increase in the number of diagnostic and treatment centres, could have contributed to the reduced fatality tendency [14].

In the period covered by this study, a lethal parasite species was identified in 400 (21.1%) deaths. These results are similar to the ones reported in other studies conducted in Colombia, where the species that caused the death in 25.7% of the cases was identified [14]. In this sense, the study is consistent with other research because it concludes that the species most frequently associated with lethality was *P. falciparum*, followed by *P. vivax*[14].

The 2013 World Malaria Report [16], mentions that worldwide *P. falciparum* continues to be responsible for the majority of malaria related deaths. However, *P. vivax* is also profiled as an important causal species for complicated malaria and malaria mortality. It should be highlighted that establishing the true magnitude for malaria mortality is difficult worldwide. The current sub-registry of information of the deceased, the difficulties to determine the exact cause of death due to difficulties accessing rural healthcare systems and the difficulties regarding the access and opportune systematization of this information are factors that complicate the registry of data in surveillance systems.

## Conclusions

Despite the above, results of this study and other studies support the idea that the control measures implemented in Colombia around the different periods have reached an important decrease of the epidemiological indicators of malaria, such as morbidity and mortality rates. However, it is important to acknowledge that, currently, Colombia is at a special moment that should aim at eliminating malaria. The improvement of the diagnosis and therapeutic methodology, the knowledge of the local transmission features, the active detection of cases and of population at risk as well as the methodologies for public health surveillance must be aspects of mandatory inclusion in the planning of future policies to optimize the control of malaria in Colombia.

## Abbreviations

ARIMA: Autoregressive integrated moving average; SIVIGILA: Public health surveillance system; DANE: National administrative department of statistics; API: Annual parasite index; AFI: Annual malaria index for falciparum.

## Competing interest

Authors declare not having any conflict of interest.

## Authors’ contribution

GC and AP conceived the study. IG and AP gathered the necessary information. AP carried-out the statistical analysis, interpreted the results and wrote the draft of the article. GC supervised the statistical analysis, interpreted results and managed the critical revision of the manuscript. IG helped interpreting and reviewing the manuscript. JP did the adaptation of the manuscript for the journal and collaborated in the realization of the discussion. All authors read and approved the final manuscript.
